# Characterising the role of the *Eucalyptus grandis SND2* promoter in secondary cell wall biosynthesis

**DOI:** 10.1186/1753-6561-5-S7-P105

**Published:** 2011-09-13

**Authors:** Jonathan Botha, Desre Pinard, Nicky Creux, Steven Hussey, Christine Maritz-Olivier, Antanas Spokevicius, Gerd Bossinger, Eshchar Mizrachi, Alexander Myburg

**Affiliations:** 1 1Department of Genetics, Forestry and Agricultural Biotechnology Institute (FABI), University of Pretoria, Pretoria, 0002, South Africa; 2 2Department of Forest and Ecosystem Science, Melbourne School of Forest and Ecosystem Science, University of Melbourne, Australia

## Background

NAC and MYB transcription factors (TFs) have been shown to play prominent roles in the regulation of plant developmental processes. Two *Arabidopsis thaliana* NAC domain TFs (AtSND2, AtSND3) and one MYB domain TF (AtMYB103) were shown to be downstream targets of two master regulators of xylem fibre cell development, NST1 and SND1 [1,2]. These TFs were able to induce the expression of a GUS reporter gene under the control of the *AtCesA8* promoter [3], indicating that they may be involved in the regulation of cellulose biosynthesis in the secondary cell walls of *A. thaliana* xylem fibres. It is hypothesized that putative orthologs of these TFs will also play important roles in regulating fibre secondary cell wall biosynthesis in woody plants such as *Eucalyptus grandis.* The transcriptional network regulating wood fibre development is uncharacterized in *E. grandis*, therefore it would be beneficial to identify upstream components of this transcriptional network in *E. grandis.* In this ongoing study we aim to identify TFs which bind to the promoter of the putative ortholog of *AtSND2* in *E. grandis* (*EgSND2*) and possibly also to the promoters of the orthologs of *AtSND3* and *AtMYB103.* In parallel, we are characterizing the detailed heterologous expression patterns of the *EgSND2*, *EgSND3* and *MYB103* promoter regions in *A. thaliana* plants. This work forms part of an effort to elucidate the transcriptional network regulating wood fibre development in *E. grandis.*

## Methods

A reverse BLAST approach was used to identify candidate orthologs of *AtSND2*, *AtSND3* and *AtMYB103* in *Eucalyptus*, *Populus* and *Vitis. In silico cis-*element analysis was performed on the promoter regions of the putative orthologs to identify previously characterised and novel *cis-*elements. The 1.5 kb regions upstream of the translational start site (TSS) of *EgSND2*, *EgSND3* and *EgMYB103* were isolated from *E. grandis* genomic DNA. The amplified fragments were cloned into pMDC162, a GUS reporter vector, and introduced into *A. thaliana* Col-O plants for heterologous GUS expression analysis. The same reporter constructs were also transformed directly into the vascular cambium of potted *E. grandis* plants to determine endogenous promoter activity by Induced Somatic Sector Analysis (ISSA, [4]). Qualitative GUS reporter analyses were performed on the *EgSND2promoter::GUS*, *EgSND3promoter::GUS* and *EgMYB103promoter::GUS* constructs in *Arabidopsis* plants at 1, 3 and 6 weeks. A 500 bp truncation upstream of the TSS of *EgSND2* was generated and cloned into the pHIS2.1 vector along with the 1.5 kb *EgSND2* promoter sequence for use in yeast one-hybrid (Y1-H) screening. Candidate proteins which bind to the cloned promoter sequences will be identified using Y1-H analysis and further characterised.

## Results and discussion

A number of previously described (e.g. [5]) and novel *cis-*elements were present in the three cloned *Eucalyptus* promoters (*EgSND2*, *EgSND3* and *EgMYB103*)suggesting regulation by an overlapping set of upstream transcription factors. The 1, 3 and 6-week GUS analyses of the *EgSND2promoter::GUS* and *EgSND3promoter::GUS* constructs revealed strong GUS expression in vascular tissues, but the GUS expression was not specific to vascular tissues as reported for the endogenous *AtSND2* and *AtSND3* genes [3]. This suggests that the 1.5 kb region upstream of the translational start site may not be sufficient for fibre-specific expression in a heterologous system. Similarly, the *EgMYB103promoter::GUS* construct was expressed in stems and leaves, in contrast to strong stem specificity reported by Zhong et al., [3] for the *Arabidopsis* ortholog. ISSA (ongoing) results of endogenous expression in *Eucalyptus* may clarify the possible regulatory divergence in these promoter sequences. We hope to soon identify and functionally annotate a number of protein candidates binding to the *EgSND2* promoter using Y1-H analysis.

## Conclusions

The *EgSND2*, *EgSND3* and *EgMYB103* promoters were found to contain common *cis-*regulatory elements, which suggests at least partial co-regulation in *E. grandis.* The 1.5 kb upstream regions of *EgSND2*, *EgSND3* and *EgMYB103* induced strong heterologous GUS expression in vascular and non-vascular tissues of *A. thaliana* suggesting either that these promoter sequences have functionally diverged in *Arabidopsis* and *Eucalyptus*, or that the 1.5 kb upstream regions alone are not sufficient to induce fibre-specific expression as previously reported in *Arabidopsis.*
